# Folic Acid Food Fortification—Its History, Effect, Concerns, and Future Directions

**DOI:** 10.3390/nu3030370

**Published:** 2011-03-15

**Authors:** Krista S. Crider, Lynn B. Bailey, Robert J. Berry

**Affiliations:** 1 The Division of Birth Defects and Developmental Disabilities, National Center on Birth Defects and Developmental Disabilities, Centers for Disease Control and Prevention, Atlanta, GA 30033, USA; Email: rjb1@cdc.gov; 2 The Food Science and Human Nutrition Department, University of Florida, Gainesville, FL 32611, USA; Email: folate@ufl.edu

**Keywords:** folic acid, flour fortification, neural tube defects, cancer, epigenetics

## Abstract

Periconceptional intake of folic acid is known to reduce a woman’s risk of having an infant affected by a neural tube birth defect (NTD). National programs to mandate fortification of food with folic acid have reduced the prevalence of NTDs worldwide. Uncertainty surrounding possible unintended consequences has led to concerns about higher folic acid intake and food fortification programs. This uncertainty emphasizes the need to continually monitor fortification programs for accurate measures of their effect and the ability to address concerns as they arise. This review highlights the history, effect, concerns, and future directions of folic acid food fortification programs.

## 1. History

### 1.1. Folic Acid and the Prevention of Neural Tube Defects

Folate is a water-soluble vitamin and includes endogenous food folate and its synthetic form, folic acid. In its naturally occurring form folate lacks stability in food storage and preparation [1]; however, folic acid is stable [1,2,3] and used for supplements and food fortification. There are many critical cellular pathways dependent on folate as a one-carbon source, including DNA, RNA, and protein methylation, as well as DNA synthesis and maintenance [4]. A number of genetic polymorphisms affect critical components of folate pathways and metabolism, and have been associated with an increased risk for NTDs [5]. However, the exact mechanism(s) by which folic acid reduces the risk of NTDs is not known and remains an active area of research.

Neural tube defects (NTDs) occur when the neural tube fails to close early in embryonic development, resulting in damage to the exposed underlying neural tissue. These birth defects can result in significant morbidity and mortality depending on the location and severity of the lesion. The most severe―anencephaly―is incompatible with life; the lower lesions observed with spina bifida cause a range of morbidities, including urinary and fecal incontinence and paralysis of the lower limbs [6].

The relationship between apparent folate deficiency and NTD occurrence was hypothesized as early as 1965 [7]. After a number of studies suggested that folic acid might reduce the risk of NTDs [8,9,10], a randomized control trial (RCT) to determine the effectiveness of folic acid supplementation in the prevention of the recurrence of NTDs was undertaken by the British Medical Research Council [11]. That RCT found that women with a previous history of a pregnancy affected by an NTD reduced their recurrence risk by 70% by taking 4000 micrograms (µg) of folic acid daily. The following year, in a Hungarian RCT, a 100% reduction in risk of a first occurrence of an NTD-affected pregnancy was found among women who took a prenatal vitamin containing 800 µg of folic acid daily [12]. 

In 1991, the Centers for Disease Control and Prevention recommended that women with a history of a prior NTD-affected pregnancy should consume 4000 µg of folic acid daily starting at the time they begin planning a pregnancy [13]. Subsequently, in 1992, the U.S. Public Health Service recommended that all women of childbearing age consume 400 µg of folic acid daily through fortification, supplementation, and diet to prevent NTDs [14]. In 1998, the Institute of Medicine (IOM) recommended that women capable of becoming pregnant should consume 400 µg of folic acid daily from fortified foods or supplements, or both, in addition to that obtained through a normal diet [15]. In 2009, the U.S. Preventive Services Task Force published updated guidelines that reinforced these recommendations [16].

Encouraging women to consume a supplement containing 400 µg of folic acid daily has limitations as a primary public health program. In the United States, up to 50% of all pregnancies are unplanned [17]. The neural tube closes early in embryonic development (28 days after conception), therefore a woman should begin folic acid supplementation ideally prior to becoming pregnant. Education campaigns encouraging women to increase their use of supplements have not been effective at reaching every high-risk population [18]. To compound the problem, a recent UK cohort study showed that women planning a pregnancy only marginally increased their compliance with health behaviors and folic acid supplement use [19]. Other countries have recommendations to prevent NTDs by improving diet or encouraging supplement use, or both, but do not have mandatory fortification programs. An evaluation of NTD trends in such countries has revealed no significant changes since recommendations were enacted [20]. It has been suggested that well-implemented mandatory fortification programs, unlike voluntary fortification programs, might help reduce disparities [21]. Given these issues, mandatory fortification programs have been implemented in many countries to maximize their effects and reduce the high costs associated with prevention programs such as education campaigns and other interventions that require behavioral change [22]. 

### 1.2. Mandatory Food Fortification Programs

Regulations for mandatory fortification of wheat flour with folic acid are currently in place in 53 countries although in many cases these regulations have not been implemented [23]. In 2006, the World Health Organization and the Food and Agricultural Organization of the United Nations published guidelines to help countries to set the Target Fortification Level, the Minimum Fortification Level, the Maximum Fortification Level and the Legal Minimum Level of folic acid to be used to fortify flour with folic acid [24]. In the United States, mandatory fortification of enriched cereal grain products with folic acid was authorized in 1996 and fully implemented in 1998 [25]. The U.S. program adds 140 µg of folic acid per 100 g of enriched cereal grain product and has been estimated to provide 100–200 µg of folic acid per day to women of childbearing age [26,27,28]. Although mandatory flour fortification programs increase folic acid intake, research has shown that they do not reach all women of reproductive age adequately [29,30]. For maximum benefit, folic acid fortification of additional food products might be needed to reach all population groups effectively [31]. The mandatory folic acid fortification level in select countries including the US is illustrated in Table 1 [32]. 

**Table 1:** Levels of folic acid fortification in countries with mandatory fortification programs.

Country	Fortification level	Date of implementation
United States [25]	140 µg/100 g	1998
Canada [33]	150 µg/100 g	1998
Costa Rica [34]	180 µg/100 g	1998
Chile [35]	220 µg/100 g	2000
South Africa [36]	150 µg/100 g	2003

## 2. Effect of Mandatory Fortification of Food with Folic Acid on NTD Prevention

### 2.1. NTDs

The original impetus for folic acid food fortification programs was to reduce the occurrence of NTDs and associated morbidity and mortality, although a number of other health effects have been postulated. For the purposes of this review, we have limited this discussion to NTD risk reduction. Folic acid fortification to reduce NTDs is considered one of the most successful public health initiatives in the past 50–75 years [32]. Studies in the United States, using various methodologies, have shown decreases of 19%–32% in the prevalence of NTDs overall since the implementation of folic acid fortification in 1998 [37,38,39,40]. Larger decreases have been found in programs that were able to capture NTD cases using prenatal information, including elective terminations [37]. Recent reports have suggested that anencephaly and spina bifida have seen similar declines, although it took additional years of fortification for the declines in anencephaly to match those of spina bifida [23,40].

Canada, South Africa, Costa Rica, Chile, Argentina, and Brazil also have reported declines in NTDs (19%–55%) since the initiation of folic acid food fortification [33,34,35,36,41,42,43,44,45,46]. The magnitude of the decrease in NTD prevalence observed in each country has been dependent on a number of factors. The greatest decline in NTD prevalence has been observed in those countries with the highest background prevalence. Other variables that have influenced the decline in NTD prevalence after fortification include the folate status of the population before fortification, the number of people consuming fortified foods, and the ability of birth defects surveillance systems to effectively ascertain the decline in prevalence of NTDs as a result of folic acid fortification. Although there were variations in the rates of NTDs among countries before fortification (10.6–17.0 cases per 10,000 live births), since folic acid fortification the decreased rates have been less variable (6.3–10.0 cases per 10,000 live births), as previously reviewed [32]. These data suggest that a prevalence of 5–6 cases per 10,000 pregnancies represents the lowest prevalence that is achievable through current folic acid fortification practices and that a proportion of the remaining NTDs are not sensitive to folic acid [23,32]. Recent evidence indicates that low maternal vitamin B12 is a significant predictor (independent of folate) of NTD risk [47].

### 2.2. Blood Folate Concentrations and the Prevention of NTDs

One way to evaluate the effect of food fortification programs is to measure blood folate concentrations among the population. Folate deficiency is defined as a serum folate concentration <7 nmol/L (~3 ng/mL) or a red blood cell folate concentration <315 nmol/L (~140 ng/mL) [15]. Homocysteine concentration may be considered a “functional” indicator of folate status in conjunction with blood folate concentrations [48]. Although the blood folate concentration needed to achieve a maximal reduction in the risk for folate-sensitive NTDs is unknown, among an Irish cohort, Daly *et al.* found that the greatest reduction in NTDs was observed at blood folate concentrations much higher than those set for folate deficiency [49]. This suggests that for NTD prevention elimination of folate deficiency is insufficient. Folic acid from multiple sources might be needed to achieve a blood folate concentration high enough for optimal NTD risk reduction [31]. 

### 2.3. Higher Folic Acid Intake Relative to Sources

In the United States, folic acid intake can come from multiple sources, including supplements, enriched cereal grain products, ready-to-eat breakfast cereals as well as other types of fortified food. A variety of foods can be fortified with folic acid under different U.S. Food and Drug Administration rules―such as ready-to-eat breakfast cereals and energy bars and drinks―as well as from the mandatory fortification of enriched cereal grain products [50]. Supplements and ready-to-eat cereals provide up to 400 μg of folic acid in pill form or per serving, respectively. 

Since the implementation of mandatory folic acid fortification in the United States, the types of population-based studies examining the potential beneficial or adverse effects of such fortification have been limited. Available studies―such as cross-sectional, ecological, and case reports―cannot be used to establish causation. Most of the concern surrounding folic acid fortification comes from studies of higher intakes of folic acid or higher blood folate concentrations, or both. Pills used in RCTs testing the effectiveness of folic acid intake on other health outcomes commonly contain 800–2500 µg of folic acid [51], and higher blood folate concentrations are associated with excess use of supplements containing folic acid [50,52]. 

The tolerable upper intake level (UL) (1000 μg/day) was established by the IOM in 1998 as one-fifth of the lowest observed adverse effect level (5000 μg/day) associated with a potential adverse outcome in early case reports (the masking of vitamin B12 deficiency anemia) [15]. Even at dosages of 15,000–100,000 μg of folic acid daily, the IOM found limited evidence of direct toxicity from folic acid [53,54,55,56,57,58]. Recent studies have shed light on the means by which the intake would exceed the UL for most individuals. In a nationally representative study from the United States, Yang *et al.* found that food fortification supplied a steady low level of folic acid intake (~138 µg/day) and only 2.7% of the adult population had folic acid intakes that exceeded the UL [50]. Moreover, the only adults with usual intakes above the UL were those who consumed more than 400 μg of folic acid daily through supplements [50,59]. Among children whose usual intake of folic acid was limited to that from enriched cereal grain products, no one’s usual intake exceeded the UL [60]. 

## 3. Concerns about Potential Adverse Effects

With any public health intervention, there are concerns about potential adverse consequences. Continued monitoring of fortification programs is critical to be able to address emerging concerns as new hypotheses are generated. Monitoring could include regulatory oversight of the flour fortification industry, surveillance of NTDs if possible, surveys of blood folate concentrations in the population, and surveillance for potential adverse effects. Assessing the sources and amounts of folic acid consumed is critical to studies because we know that fortification is a relatively small contributor to higher levels of daily folic acid intake. Folic acid has been used successfully for more than 40 years and throughout its use there have been concerns about its safety. In this review, we address some of the old and newly emerging concerns. 

### 3.1. Masking of B12 Deficiency Anemia

Historically, concerns surrounding folic acid use have focused on the possibility that folic acid could mask the anemia caused by vitamin B12 deficiency. Early case reports (1940–1960) suggested ≥5000 µg of folic acid daily could mask a vitamin B12 deficiency by preventing the development of anemia. In turn, this could delay the diagnosis of an underlying vitamin B12 deficiency and thereby allow vitamin B12 deficiency-associated neuropathies to progress [15]. It is recognized that the diagnosis of a vitamin B12 deficiency and/or response to treatment should be dependent on a series of vitamin B12 blood status indicators and not solely on hematological indices which may not be reliable [15]. The IOM concluded in 1998 that there was “no clear evidence of folate-induced neurotoxicity in humans” [15]. Several studies both before and after fortification have examined this issue and concluded that, at current recommended intakes, there is little evidence of masking or exacerbation of neuropathies [61,62,63,64]. 

### 3.2. Cancer and Epigenetic Changes

Many studies, as reviewed, have shown that a diet high in folate (from fruits and leafy green vegetables) is associated with lower risks of many types of cancer [65]. Recently, focus has shifted to the possibility that folic acid intake might lead to changes in epigenetic patterns. Epigenetics is the study of heritable changes that do not involve changes in DNA sequence. Folate is used by two different metabolic pathways―DNA synthesis and DNA methylation (an epigenetic modification)―as a source of one-carbon methyl groups [4]. Epigenetics has been hypothesized to play a key role at the interface of gene–environment interactions and might help to explain different health outcomes (e.g., birth defects, cancer, and mental health) occurring among those with similar genetic backgrounds [66,67,68]. 

As a result of the fact that folate is a source of the methyl group for DNA methylation and that DNA methylation is a ubiquitous regulator, there are countless biologically plausible hypotheses of how folic acid might affect any disease of interest, either positively or negatively. Most of the concern has surrounded cancer, most likely because the field of epigenetics has been studied largely in the context of tumorogenesis [69,70,71,72,73,74]. There are clear DNA methylation pattern changes in tumors [73,74,75]. It has been suggested in a recent review that folic acid might prevent some cancers and might promote other neoplasias [76]. It has been hypothesized that early exposure to folic acid might prevent tumors through the provision of enough methyl groups to maintain proper methylation patterns and repair of DNA. In contrast, after the development of tumors, higher intake of folic acid might promote growth of existing tumors [76]. However, a very recent meta-analysis of the RCTs of the effects of B vitamins on 37,485 individuals with existing cardiovascular disease showed no increased risk of cancer incidence (relative risk (RR) 1.05, 95% confidence interval (CI) 0.98–1.13), cancer mortality (RR 1.00, 95% CI 0.85–1.18) or all-cause mortality (RR 1.02, 95% CI 0.97–1.08) [51]. Since the implementation of mandatory folic acid fortification in the United States in 1998, both incidence and mortality of colorectal cancer have continued to decline [77]. 

In 2008, the European Food Safety Authority (EFSA) convened a working group to consider whether there was enough evidence to recommend a full risk assessment to determine whether folic acid was causing cancer, especially colorectal cancer. The EFSA concluded that, “there are currently insufficient data to justify such an assessment and that current evidence does not show an association between high folic acid intakes and cancer risk but neither do they confidently exclude a risk” [78].

There are a number of knowledge gaps that need to be filled before we know if folic acid affects disease risk through DNA methylation in humans. The “normal” patterns of DNA methylation across the genome are unknown, as are the “normal” levels of variation among and between individuals and populations. The National Institutes of Health has an initiative to map the epigenome that is modeled on the Genome Project to begin to describe DNA methylation. It also is unknown if DNA methylation patterns among humans can be altered by folic acid or other micronutrients, or both, or if the patterns change by the timing of exposure (e.g., different response among adults *vs.* among children or fetuses). Animal models have shown that it is possible to alter the prenatal exposures to one-carbon sources (folic acid, choline, betaine, *etc.*) and affect DNA methylation at a specific locus and that this can affect the phenotype of the offspring regardless of the adult animal’s diet [79]. Currently, there is no evidence of this effect among humans. The interaction of folic acid and epigenetics will continue to produce hypotheses that will need to be tested carefully. 

### 3.3. Unmetabolized Folic Acid

Folic acid normally is reduced to tetrahydrofolate following uptake by the liver [80]. If the body’s ability to reduce folic acid is exceeded, unmetabolized folic acid will be found circulating in the blood. One experimental study suggested that unmetabolized folic acid would be found after consuming a bolus of >200 μg of folic acid [81]. Intakes exceeding this threshold would be common through the use of supplements or fortified foods such as breakfast cereals [82], but unlikely would be reached through intake of folic acid from mandatory U.S. fortification levels alone [81,83,84,85,86,87]. Because folic acid has been a long-standing component of over-the-counter supplements and prenatal vitamins, if looked for, unmetabolized folic acid would have been found among a large proportion of the U.S. population for decades. Only recently has the laboratory equipment needed to measure circulating unmetabolized folic acid become available. Unmetabolized folic acid has been found among many groups examined―from older U.S. adults [87] to the cord blood from newly delivered infants [88]. It has been hypothesized that unmetabolized folic acid is related to cognitive impairment among seniors [89], although the findings might have been confounded by patients with pernicious anemia. Currently, there are no definitive studies that have found health effects from exposure to unmetabolized folic acid.

## 4. Systematic Reviews of Potential Beneficial and Adverse Effects Prior to the Implementation of Folic Acid Fortification Programs

The food safety agencies in a number of countries considering mandatory folic acid fortification have considered the potential that folic acid may have both beneficial and adverse effects. Among these agencies are the UK Food Standards Agency (FSA) [90], the Food Safety Authority of Ireland (FSAI) [91], Food Standards Australia New Zealand (FSANZ) [92], and the Health Council of the Netherlands [93]. The recommendation to approve folic acid fortification has been the consensus decision presented in final reports from these countries. However, for a variety of reasons, to date only Australia has completed implementation of its program.

### 4.1. Experiences in the United Kingdom

In 2006, the Scientific Advisory Committee on Nutrition (SACN) of the UK FSA recommended that mandatory fortification with folic acid should proceed, together with controls on the intake of folic acid from voluntarily fortified foods [90]. Then in 2007, the SACN was convened at the request of the UK FSA to review potential adverse effects of folic acid on colorectal cancer risk because of two papers published earlier that year. In 2009, after an extensive review, the panel concluded that there currently were insufficient data to support the concerns that folic acid fortification promoted cancer and announced its decision to support its previous recommendation for mandatory folic acid fortification [94].

### 4.2. Experiences in Ireland

In 2006, the FSAI recommended that mandatory fortification with 120 µg of folic acid per 100 g of bread should begin without changing the existing practice of voluntarily fortifying foods with folic acid [91,92,93,94,95]. However, in 2008, the FSAI implementation group reported to the Department of Health and Children that mean intake of folate in Ireland had increased to 90 µg daily, primarily from the intake of folic acid from voluntarily fortified foods, of which the FSAI documented more than 200 individual kinds of foods [96]. In 2009, the FSAI announced that because (1) women of childbearing age were receiving 30% more folate in their diet than they were 3 years earlier (the source of which was mostly from voluntary fortification) and (2) the incidence of NTDs in Ireland had been reduced to 9.3 per 10,000 livebirths, there would be limited benefit to public health to require mandatory folic acid fortification at that time [97]. This decision will be open to reconsideration as additional information becomes available on potential adverse outcomes [97]. Additionally, because previous studies have shown that voluntary fortification likely does not reach all subpopulations of women of childbearing age who are at risk [21] and given that NTD prevalences of 5–6 per 10,000 livebirths have been achieved among other populations [98], there might be room for further reductions in folic acid-preventable NTDs in Ireland.

### 4.3. Experiences in New Zealand and Australia

In 2007, FSANZ recommended that Australia and New Zealand implement mandatory programs to fortify bread with folic acid. Both governments agreed to implement this plan by September 2009 [92]. However, in June 2009, bakers in New Zealand began a campaign to stop the plan [99]. The media campaign funded by the Baking Industry Association of New Zealand raised many concerns in the public’s mind as to the safety of folic acid fortification. As a result, the plan for mandatory fortification of bread in New Zealand was put on hold for 3 years [100]; Australia, however, implemented mandatory fortification as scheduled in September 2009.

## 5. Future

Existing folic acid food fortification programs have reduced significantly the number of pregnancies affected by NTDs and the associated morbidity and mortality. In the future, new hypotheses will be generated that will need exploration and testing, such as concerns over the possibility of epigenetic changes. Careful monitoring of existing and proposed programs will enable the scientific community to evaluate blood folate concentrations needed for NTD prevention, evaluate and respond appropriately to concerns as they arise, and document the benefit of these public health programs. As with any public health program, it is important to revisit recommendations regularly as additional information becomes available. A significant portion of the estimated 300,000 NTDs worldwide that occur yearly are preventable by the consumption of folic acid and continue to be a great public health burden globally [32].
